# Medical Seed-Time—Professional Omnibuses

**Published:** 1831-01-01

**Authors:** 


### Periscope;

OR,

### CIRCUMSPECTIVE REVIEW.

"Ore trahit quodcunque potest, atque addit acervo."

### I.

### Medical Seed-time. Professional Omnibuses.

The numerous labourers in the fields of medical science are now in full employment; thanks to the non-introduction of steam and machinery among the means of infusing sound medical and chirurgical knowledge into the minds of our youth. If the Lord shall vouchsafe his blessing upon the seed thrown into the ground this season, we may anticipate a more plentiful crop than was before reaped on these happy shores.

The list of Ouvriers in this important field, as seen in fifteen closely printed pages of the Lancet—or even in the advertisement sheet of our last number, must appear "prodigious," when we consider that "Modern Babylon" alone, is the theatre of their operations! As the number of teachers, in medicine, as in other branches of knowledge, must bear some ratio to the pupils who present themselves for tuition, it is not easy to account for the astounding increase of both classes, on any calculation connected with the increment of population in this country. We were bewildering our brains with attempts to solve this enigma, when a drive along the New-road, from the Yorkshire Stingo to the Islington Gate, the other day, let in some light on the subject. We think we may safely affirm that the *traveller*, on this short and rapid journey of two miles or less, runs more risk of life and limb, than between London and Seringapatam, whether the intervening space be passed by land or by water! The Insurance Companies should look to this, and enjoin an extra premium on life policies between Paddington and the Bank. The old Stages—five or six in number, instantly reminded us of the medical schools of London, some thirty or thirty-five years ago—while the Omnibuses, flying rather than running along this road, represented very accurately, the omnium gatherum seminaries for medical education now prevailing in Modern Babylon. In despite of the "bodily fear" into which we were thrown, on this journey, lest we should be *run over*, or, at all events, *over-run*, in the "March of Intellect," we had time to remark that many of the Omnibuses were proceeding at a rapid rate, and with every badge of *external* decoration and triumph, although a peep into the interior of some of them, disclosed to us a sad vacuity! Still, with every allowance for bad fares and bad times, there can be no doubt that the number of passengers along the road above-mentioned, has been quadrupled during the last ten years, and that without a corresponding increase of the population. The unavoidable inference is, that the Omnibuses have *created* passengers.

We think that, by a parity of reasoning, the prodigious increase of medical schools—or as they may be called, Medical Omnibuses, has been productive of an increase in the number of medical passengers along the path of professional education.

It is not our intention to enter into a categorical delineation of the medical Omnibuses of this Metropolis—a cursory glance at a few of the principal will be sufficient.

1st. A highly respectable Stage has taken in fares for nearly a century, at

Hyde Park Corner. It is called the ST. GEORGE, and was long celebrated as a favourite vehicle of the renowned John Hunter. Although a thriving concern, it was lately deemed advisable, in accordance with the "MARCH OF INTELLECT," to break it up, and replace it by one of the most splendid OMNIBUSES which now grace the Metropolis. As it never leaves the vicinity of the parks and the palace, it always enjoys *fresh air;* and, as the majority of the proprietors are "learned Thebans," from Oxford and Cambridge, it is the most *Aristocratic* Omnibus in London. The passengers, who are denominated, "par excellence," WEST-ENDERS, are of a very superior description, and look down, with some degree of contempt, on their cockney compatriots.

2d. The "MIDDLESEX OMNIBUS" has never been a very lucrative speculation. As it does not carry its passengers the whole length of their journey, it is obliged to employ certain "branch-coaches," and even flys, which occasion some loss of time, and considerable inconvenience. A proposition was lately made to incorporate it with a new and popular Omnibus, got up by a JOINT-STOCK COMPANY, in the neighbourhood; but there was a leaven of aristocratic blood in the proprietors of the old vehicle, which repelled the proffered alliance—much to the disadvantage of both concerns!

3d. The "POPULAR or UNIVERSAL OMNIBUS," to which allusion has just been made, was built on a broad foundation, uniting both learning and science, under the auspices of a celebrated "SCHOOL-MASTER," and a noted "MECHANIC," who were, alternately or conjointly, to drive the machine. The cap of liberty—at least "liberty of conscience," surmounted the gorgeous receptacle; but the immense number of proprietors, who naturally claimed a voice in the direction, proved that "in multiplicity of counsels" there is *not always* wisdom! The "Chamber of Deputies"—like some other Chambres, is by no means popular—and an OMNIBUS constructed of excellent materials, and calculated to accommodate an unlimited number of passengers, has not, as yet, made much return of profits to the share-holders. If democracy, like tyranny, does not carry within itself the principles of dissolution, the "UNIVERSAL OMNIBUS" may yet prove one of the most profitable speculations —for the public if not for the proprietors—which Modern Babylon has hitherto engendered. There is a link wanting in the chain of its appointments —perhaps more than one link—which may prove fatal, if not soon supplied. The want of those perpetual or free admissions for life, purchaseable at other offices, was a sad error in the proprietors. Many people like to have the liberty of free travelling, though they never go beyond their own firesides! *Verbum sat.*

4th. In a very opposite and different quarter of this wide-spread Metropolis, near the banks of FATHER THAMES, and amidst the din and bustle of countless multitudes, rushing to and fro, like the tumultuous waves of a troubled ocean, stands one of the oldest constructions of the kind in this country. It was originally set up by Monks, for the conveyance of bigotted pilgrims to the shrine of THOMAS A BECKET at Canterbury, and thence received the name of the ST. THOMAS, which it still retains. Subsequently it was converted to a much more useful purpose, as a vehicle for sick and wounded of every description, whether booked for the PORT of HEALTH or the banks of the Styx.

As there will always be rivalry in the most pious pursuits—even in the building of churches and the lighting up of the intricate road to heaven, so ST. THOMAS was destined to see a formidable competitor set up directly opposite to him, in the very same street. There was more wisdom in those days, however, than in ours. The two companies soon coalesced, and a colossal DOUBLE COACH or MEDICAL DILIGENCE entitled the UNION, was the result. It drove a most lucrative trade for centuries, and in this prototype of modern OMNIBUSES, might be seen daily taking their seats for a drive to the temple of fame and fortune, a Cheselden and a Pott—a Cline and a Cooper—a Babington and a Curry, *cum multis aliis*. But perpetual unanimity is not to be expected on earth. Only a very few years ago, by some mismanagement or negligence of the drivers, one of the wheels of the UNION went into a rut, and the overloaded vehicle parted right in the middle! Various *skilful* engineers and architects exerted themselves to *re-unite* the two bodies—but all in vain. There was no alternative but that of running the two coaches separately, and with distinct interests. One advantage or accommodation, however, was still left open to the public. A passenger who booked himself at either office, was entitled to go by whichever Omnibus he pleased—or, if he had the power of dividing himself into two halves, as the original UNION had done, he might travel by the two vehicles at one and the same time.

5th. The last of the great professional Omnibuses which we need notice, takes its stand near SMITHFIELD Market, and is one of no mean note. During the first quarter of the present century, it attracted an immense number of passengers, chiefly on account of a humorous and eccentric DRIVER, whose jibes and jokes afforded infinite entertainment to the medical ELEVES of BARTHOLOMEW-CLOSE, Cock-lane, Little Britain, Falcon-square, Barbican, Silver Addle, Saffron-hill, Cow-cross, Grub-street, Swan Alley, Wilderness Row, Moorfields, and numerous other localities well known to fame—or at least to ILL-FAME. The merry coachman has now retired; but he has left several successors, who conduct the OMNIBUS with considerable skill, and we believe it is one of the most productive concerns of the kind in this metropolis.

Besides these regular and established OMNIBUSES for the conveyance of medical passengers, there is a swarm of minor vehicles—vans, flys—go-carts, &c. which may be seen plying for fares at the corner of almost every street in this great city! How the proprietors contrive to pick up a livelihood, is an enigma to all but themselves. It is true they go somewhat under the established fares, and many of them profess to take their passengers through intricate lanes and alleys, by which the journey is said to be greatly shortened. But then it is notorious that some of them annually break down, to the loss and danger of their inmates and outmates—while others, in the search after near-cuts, arrive at the goal much later than the regular stages.

---

